# Single-port laparoscopic cholecystectomy with indocyanine green fluorescence in a patient with gallbladder calculi and congenital thoracoabdominal visceral heterotaxy: A case report

**DOI:** 10.1097/MD.0000000000047271

**Published:** 2026-01-23

**Authors:** Dake Liu, Weibo Liu, Jianbin Gu, Fei Xue, Yubin Zhang, Jionghui Fu

**Affiliations:** a Department of General Surgery, Shijiazhuang People’s Hospital, Shijiazhuang, Hebei, China.

**Keywords:** case report, cholelithiasis, indocyanine green, single-port laparoscopy, visceral heterotaxy

## Abstract

**Rationale:**

Congenital thoracoabdominal visceral heterotaxy (CTVH) is a rare congenital anomaly characterized by complete reversal of thoracic and abdominal organ positions. This condition poses significant challenges in surgical planning due to altered anatomical landmarks, particularly in the Calot triangle region, which is critical for cholecystectomy. Laparoscopic surgery, particularly single-port laparoscopy combined with indocyanine green (ICG) fluorescence navigation, has emerged as a promising approach to address these anatomical complexities while offering the advantages of minimally invasive surgery and improved cosmesis.

**Patient concerns:**

A 67-year-old Asian Chinese male patient with a history of hypertension, type 2 diabetes mellitus, and cerebral infarction presented for management of gallbladder calculus. The patient had been aware of his CTVH since childhood, characterized by the liver being on the left side and the spleen on the right. Over the past 3 years, regular follow-up ultrasound examinations revealed progressive enlargement of gallbladder calculus, accompanied by intermittent episodes of upper abdominal pain and inflammatory symptoms.

**Diagnoses:**

The patient was diagnosed with gallbladder calculus based on ultrasound findings. Imaging also confirmed the presence of CTVH, with reversed thoracic and abdominal organ positions, further complicating surgical planning.

**Interventions:**

The patient underwent a single-port laparoscopic cholecystectomy with ICG fluorescence navigation. The fluorescence imaging provided enhanced visualization of the Calot triangle, enabling precise identification of the cystic duct and artery, thereby improving surgical safety and reducing the risk of complications. The single-port approach, utilizing a single small umbilical incision, minimized surgical trauma and ensured an aesthetic outcome.

**Outcomes:**

The surgical procedure was smoothly and successfully completed, with no notable postoperative adverse reactions observed during the patient’s hospital stay. Upon a one-week follow-up, the patient reported no postoperative complications.

**Lessons:**

This case demonstrates the successful combined application of single-port laparoscopic surgery and ICG fluorescence navigation to manage gallbladder calculus in a patient with CTVH. The fluorescence navigation enhanced safety by improving identification of critical structures, while the single-port approach provided a minimally invasive and cosmetically favorable option. This highlights the potential of advanced imaging and minimally invasive techniques in addressing complex congenital anomalies.

## 1. Introduction

Congenital thoracoabdominal visceral heterotaxy (CTVH) is a rare congenital anomaly characterized by the complete reversal of the normal positioning of thoracic and abdominal organs,^[1]^ affecting approximately 1 in 100,000 to 250,000 individuals.^[2,3]^ This condition often involves the liver being located on the left side and the spleen on the right, contrary to the typical anatomical arrangement. Patients with CTVH may present with additional congenital anomalies affecting systems such as the cardiovascular, respiratory, and genitourinary systems, further complicating clinical management.^[4,5]^

In the context of surgical interventions, CTVH poses unique challenges due to altered anatomical landmarks.^[6]^ Surgical procedures, particularly laparoscopic surgery, require precise anatomical orientation, which can be significantly hindered by the reversed intra-abdominal structures.^[7]^ The risk of intraoperative complications in such patients is higher, such as bile duct injury or unintentional organ injury. Therefore, advanced surgical techniques and imaging modalities are often required to ensure safe and effective outcomes.

Laparoscopic surgery, especially single-port laparoscopy, has emerged as a promising approach for minimally invasive interventions in complex anatomical scenarios.^[8,9]^ Single-port laparoscopic surgery (SPLS) utilizes a single small incision, often through the umbilicus, to introduce all surgical instruments and the laparoscope. This technique minimizes postoperative pain, reduces the risk of incisional hernias, and enhances aesthetic outcomes.^[10]^ Furthermore, the integration of fluorescence navigation using indocyanine green (ICG) has significantly improved the precision of intraoperative identification of critical structures, such as the cystic duct and artery.^[11]^ ICG fluorescence navigation works by introducing the fluorescent agent intravenously, which is taken up by the liver and excreted into the bile.^[12]^ This allows for real-time visualization of bile ducts and the cystic triangle during surgery. In the context of CTVH, where the anatomical orientation is reversed, this technology becomes particularly valuable. It enables surgeons to accurately identify and dissect critical structures, thereby reducing the risk of complications associated with misidentification.

## 2. Case report

### 2.1. Timeline with relevant data from the episode of treatment

**Figure 1.**
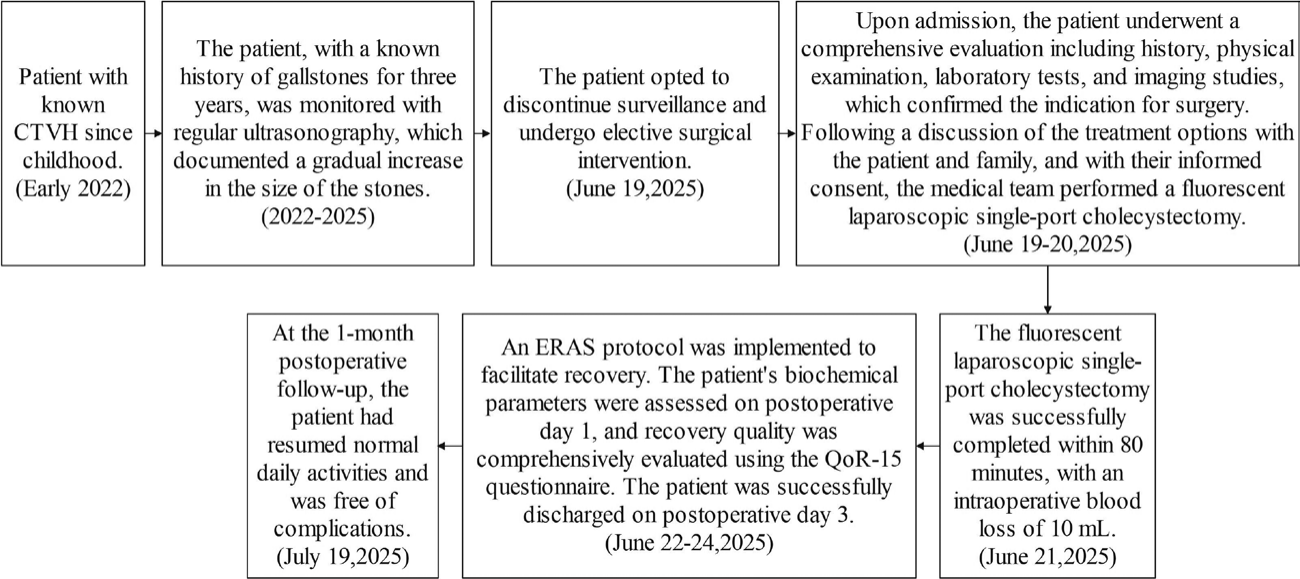
Timeline with relevant data from the episode of treatment. (A) denotes the heart, (B) denotes the stomach, (C) denotes the liver, (D) denotes the pancreas, (E) denotes the duodenum, and (F) denotes the gallbladder. The arrows indicate the gallstones.

The timeline of the patient’s treatment course is summarized in Figure 1.

### 2.2. Patient history

A 67-year-old Chinese male patient presented to our hospital with a 3-year history of gallbladder calculus detected through regular follow-up ultrasound examinations. The patient had no significant symptoms, such as upper abdominal pain, nausea, bloating, or low-grade fever. However, the gradual enlargement of the gallbladder calculus was noted during routine checkups, prompting the patient to seek surgical intervention.

The patient had a medical history, including hypertension, type 2 diabetes mellitus, and a history of cerebral infarction. For hypertension, he was taking “Indapamide” and “Nifedipine” orally, with well-controlled blood pressure. For type 2 diabetes mellitus, he was regularly using “Human Mixed Protamine Insulin Injection” at a dose of 10 IU per injection twice daily. He also had a history of old cerebral infarction, with residual numbness in the left upper and lower extremities but no significant limitation in mobility. He denied any history of alcohol or substance abuse and had no known drug allergies. His family history shows no evidence of predisposing factors, congenital anomalies, or gastrointestinal diseases.

### 2.3. Physical examination

Upon admission, the patient’s vital signs were within normal limits: a blood pressure of 123/77 mm Hg, heart rate of 70 beats per minute, respiratory rate of 19 breaths per minute, and a body temperature of 36.3°C.

The abdomen was soft and non-tender, with no evidence of rebound tenderness or muscle guarding. The liver and spleen were palpated in their atypical positions due to the patient’s CTVH. The liver was located on the left side, and the spleen on the right side, with no signs of hepatosplenomegaly. Bowel sounds were normal and symmetric bilaterally.

Cardiac auscultation revealed a regular rhythm without murmurs, rubs, or gallops. Respiratory examination showed clear lung fields bilaterally with no evidence of wheezing, crackles, or diminished breath sounds.

### 2.4. Imaging studies

Upon hospital admission, the patient underwent a chest and upper abdominal CT scan, which provided critical insights into both the anatomical and pathological aspects of his condition. The CT scan confirmed the presence of CTVH, with the liver located on the left side and the spleen on the right side. This finding was consistent with the patient’s known diagnosis of CTVH.

**Figure 2.**
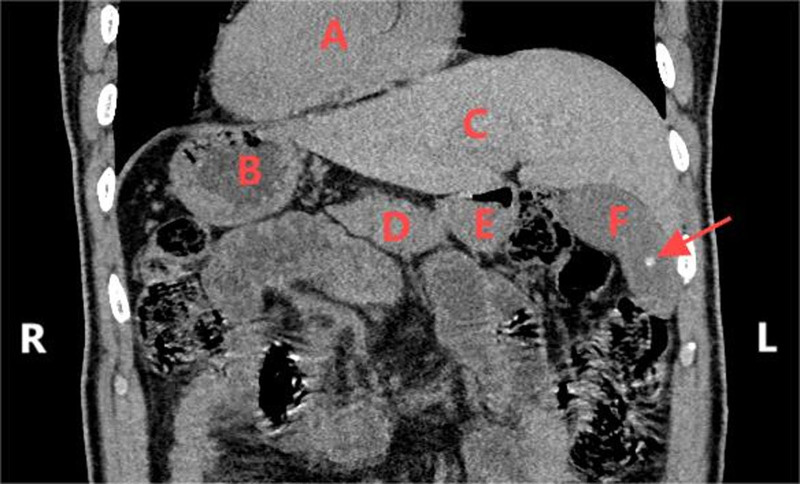
**Computed tomography** scan results upon admission.

The imaging also revealed gallbladder calculi, confirming the presence of gallstones. Additionally, the CT scan demonstrated cholecystitis, as evidenced by gallbladder wall thickening and pericholecystic inflammatory changes. The cystic duct was noted to be mildly dilated, further supporting the diagnosis of acute cholecystitis. Importantly, the CT scan ruled out any complications such as choledocholithiasis, bile duct obstruction, gallbladder perforation, or abscess formation. The specific findings are detailed in Figure 2.

These findings were instrumental in confirming the diagnosis and guiding preoperative planning. The CT scan not only highlighted the gallbladder abnormalities but also provided a detailed roadmap of the patient’s unique anatomy, which was crucial for the successful execution of the planned single-port laparoscopic cholecystectomy with ICG fluorescence navigation.

### 2.5. Laboratory biochemical testing

Comprehensive laboratory biochemical testing was conducted to assess the patient’s overall health status and confirm the diagnosis. The complete blood count revealed a white blood cell count of 6.57 × 10^9^/L, indicating no evidence of acute inflammation. The hemoglobin level was 141.90 g/L, within the normal range, and the platelet count was 128 × 10^9^/L. The biochemistry panel showed a normal alanine aminotransferase level of 13 U/L, confirming no liver dysfunction. Other liver function tests, including aspartate aminotransferase, alkaline phosphatase, and total bilirubin, were all within normal limits.

Coagulation studies, including INR, prothrombin time, and activated partial thromboplastin time, were all within normal limits, ensuring safe surgical conditions. Urinalysis results were unremarkable, with no evidence of proteinuria, hematuria, or pyuria.

These findings confirmed the absence of acute inflammation and liver dysfunction, supporting the patient’s readiness for surgery. The normal coagulation parameters further assured safe surgical conditions, and the overall biochemical profile indicated good health and appropriate preoperative status.

### 2.6. Surgical equipment

In this study, a near-infrared fluorescence imaging system developed by Beijing Precision Digital Medical Technology Co., Ltd. was utilized for intraoperative cholangiography. The fluorescent dye employed was ICG for injection, produced by Dandong Yichuang Pharmaceutical Co., Ltd., with a specification of 25 mg (Drug Approval Number: H20055881). Additionally, a disposable multi-channel single-port laparoscopic puncture device (Production Number: 104279) supplied by Mindray Medical, based in Shenzhen, China, was used. This device contained 4 operating ports: one 12-mm port for the laparoscope, one 10-mm port, and 2 5-mm ports as the main operating channels.

### 2.7. Surgical procedure

The surgical procedure was performed by a team of experienced hepatobiliary surgeons with expertise in laparoscopic and minimally invasive surgery techniques. The patient was placed under general anesthesia, and intraoperative monitoring was maintained throughout the procedure.

Regarding the dosing regimen of ICG, under sterile conditions, 25 mg of ICG powder was reconstituted with 10 mL of sterile water for injection, yielding a 2.5 mg/mL ICG solution. Inject 1 mL of this solution into the patient’s peripheral vein 0.5 to 2 hours prior to surgery.^[13]^

**Figure 3.**
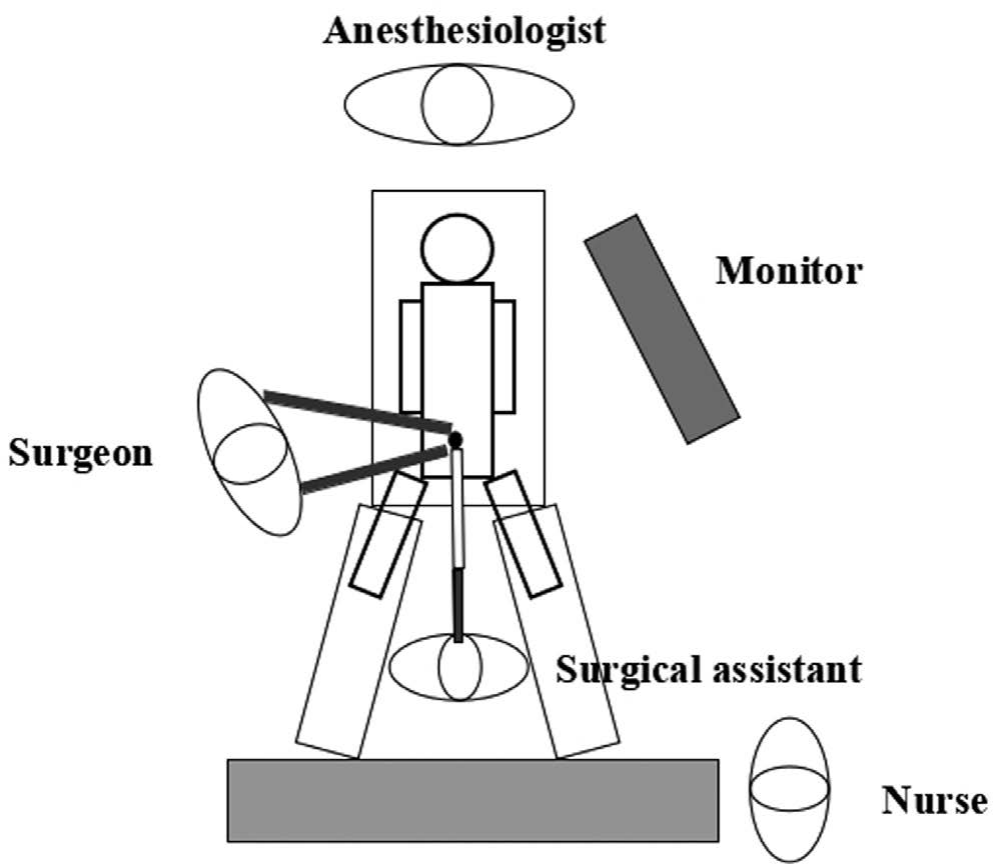
Location diagram of medical and nursing personnel during surgery. (A and B) Depict the stepwise process of making an incision above the navel and sequentially accessing the abdominal cavity during the surgical procedure; (C) Demonstrates the placement of the incision protector; (D) Illustrates the usage of a disposable multi-channel single-port laparoscopic puncture device. This instrument contains 4 operating ports: one 12 mm port (indicated by a red arrow), one 10 mm port (indicated by a blue arrow), and 2 5 mm ports (indicated by green arrows). The 12 mm port is primarily used for laparoscopic observation, while the 2 5 mm ports serve as the main operating ports. Additionally, the device includes a carbon dioxide inlet port and an exhaust port.

Due to the CTVH, the surgical team required adjustments to the surgical techniques during the procedure. The specific abdominal anatomy and surgical approach are shown in Figure 3.

**Figure 4.**
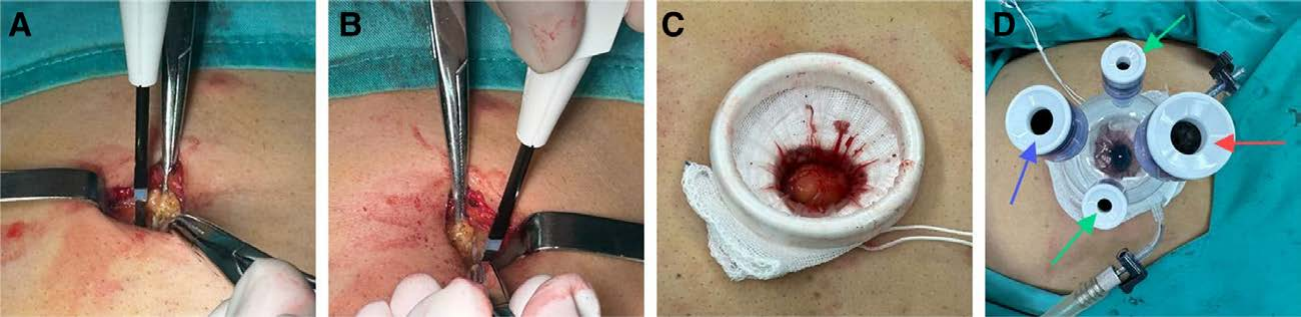
Placement procedure of the disposable multichannel single-port laparoscopic device. (A) Depicts the intra-abdominal condition of a patient undergoing laparoscopy. Label a denotes the right lobe of the liver, label b denotes the falciform ligament, label c denotes the left lobe of the liver, label d denotes the stomach, and label e denotes intra-abdominal adhesions. (B) Depicts intra-abdominal adhesions in the abdominal cavity. (C) Illustrates the anatomy of Calot triangle in white light imaging mode. (D) Depicts the anatomy of Calot triangle in fluorescence mode, where label a denotes the liver, label b denotes the common hepatic duct, label c denotes the cystic duct, label d denotes the common bile duct, and label e denotes the gallbladder.

**Figure 5.**
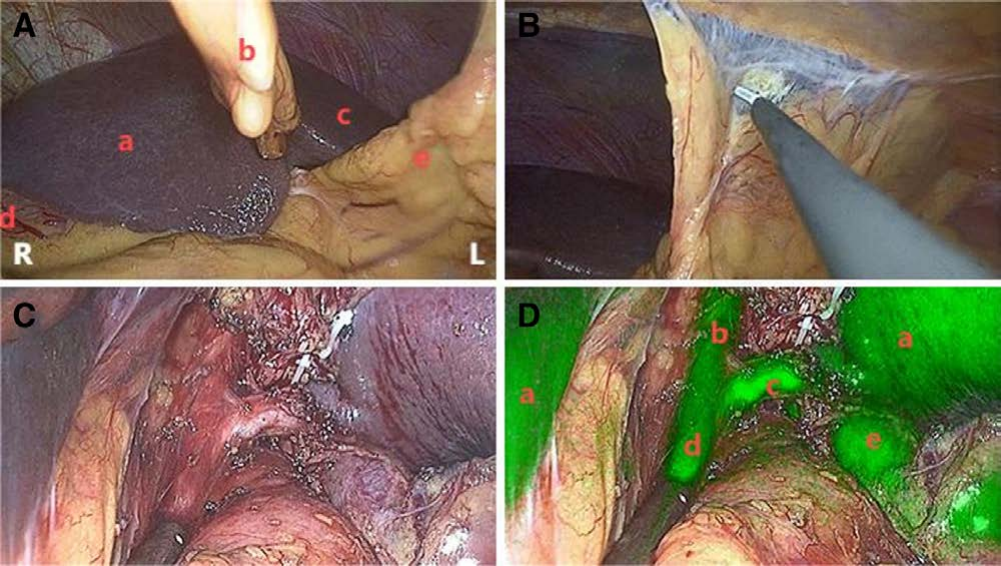
Intraoperative laparoscopic findings. (A) Depicts the real-time scenario of intradermal suturing of the incision during surgery; (B) Illustrates the appearance of the patient’s incision 3 d after surgery.

The specific steps of the surgery are:

(1) The umbilical area was disinfected and draped with sterile surgical towels, preparing the site for the single-port laparoscopic setup. A 1.5 to 2.0 cm incision was made at the umbilical region, slightly above the umbilicus. The abdominal cavity was accessed through this incision, and a single-port laparoscopic system was introduced. A pneumoperitoneum was established with carbon dioxide insufflation, maintaining an intra-abdominal pressure of 12 mm Hg to create a working space within the abdominal cavity. The specific process can be seen in Figure 4.
(2) Abdominal exploration confirmed the diagnosis of chronic cholecystitis. Consistent with the known CTVH, the liver was situated in the left upper quadrant, with the spleen located on the right. The gallbladder was found adherent to the left upper abdominal wall and the transverse colon. After careful lysis of these adhesions, the gallbladder, measuring approximately 8 × 3 × 2 cm, was exposed and showed signs of congestion and edema. The initial challenge was to identify the anatomical landmarks of the Calot triangle within this completely mirrored and adhesional anatomy.
(3) To navigate this complex anatomy, we utilized ICG fluorescence cholangiography. Following intravenous administration of 2.5 mg ICG, the extrahepatic biliary structures were clearly visualized under near-infrared fluorescence. This was particularly crucial for defining the spatial relationships within the Calot’s triangle. The cystic duct and artery were successfully identified emanating from the left-sided structures, confirming the mirrored anatomy. The ICG guidance was instrumental in distinguishing these critical structures from the surrounding adhesions and confirming their course before any dissection.
(4) The cystic triangle was carefully dissected, and the cystic artery was identified, clipped with absorbable clips, and divided. The cystic duct was isolated and managed, with the distal end treated with electrocautery. Adhesions between the gallbladder and adjacent structures were carefully divided with electrosurgical instruments using straight dissectors and graspers to fully expose the cystic neck for accurate dissection.
(5) The gallbladder was separated from the liver bed along the anatomical plane, with minimal oozing at the gallbladder bed being controlled using bipolar electrocautery. The cystic neck was opened, confirming no bile duct injury. The proximal end of the cystic neck was doubly clipped with absorbable clips, maintaining a margin of 0.5 cm from the common bile duct. The gallbladder was then completely resected and removed through the umbilical incision.
(6) The abdominal cavity was thoroughly inspected for any intra-abdominal bleeding or bile leakage, ensuring no complications. The surgical site was closed in a layered manner, and the umbilical incision was carefully sutured with absorbable sutures. Postoperative monitoring confirmed stable vital signs and no immediate complications. The specific situation during the operation is shown in Figure 5.

The operation lasted about 80 minutes with minimal blood loss of 10 mL.

### 2.8. Postoperative management

We developed an evidence-based enhanced recovery after surgery program that incorporated multimodal postoperative care for all patients.^[14,15]^ For this elderly patient with multiple comorbidities and a complex anatomical variation, our enhanced recovery after surgery protocol was tailored to ensure safety, with discharge occurring after a joint senior evaluation on the morning of the third day, confirming all physiological recovery milestones were met. Vital signs were continuously monitored until clinical stability was achieved. As part of our protocol for monitoring elderly patients with comorbidities after surgery, routine blood tests were conducted on the first postoperative day to assess complete blood count, liver, and kidney function. This baseline assessment aims to promptly identify any subclinical abnormalities in inflammation, electrolytes, or organ function, thereby ensuring close monitoring of the recovery trajectory and overall condition. Postoperatively, we ensured the timely administration of fluids, electrolytes, and energy, while also providing appropriate analgesia as needed. Patients were encouraged to mobilize early to optimize recovery and minimize complications. A dedicated nurse will be responsible for continuously monitoring and evaluating the patient’s postoperative recovery using the QoR-15 evaluation scale, as per established protocols.^[16]^

On the first postoperative day, blood tests and liver and kidney function were reevaluated. The white blood cell count was 11.71 × 10^9^/L, neutrophils were 9.90 × 10^9^/L, and serum CRP was 8.10 mg/L, with no other significant abnormalities. No other significant abnormalities were noted in liver and kidney function tests. The patient’s recovery was evaluated using the QoR-15 quality of life scale, demonstrating a score of 112 points. The patient indicated an acceptable mental state and sleep quality, with no recorded instances of flatus, fever, nausea, or vomiting.

**Figure 6.**
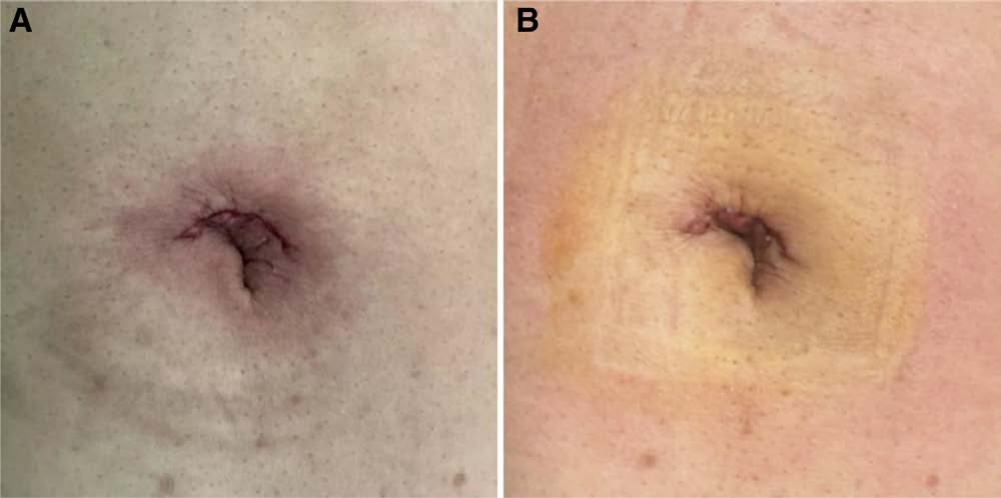
Patient’s surgical incision condition.

On the second postoperative day, the patient exhibited good spirits and reported no discomfort, with no other notable abnormalities observed. Recovery was assessed using the QoR-15 quality of life scale, which yielded a score of 128 points, reflecting a 16-point improvement from the first postoperative day. More information about the patient’s surgical incision is shown in Figure 6.

The patient was discharged on the third day after surgery. The postoperative pathology report confirmed chronic cholecystitis with glandular hyperplasia and gallstones.

At the 1-week follow-up, the patient indicated complete resolution of symptoms with no evidence of surgical complications, having achieved full recovery and resumed normal activities.

## 3. Discussion

The successful management of this patient with gallbladder calculus and CTVH highlights the importance of advanced surgical techniques in addressing complex anatomical challenges. This case demonstrates that integrating ICG fluorescence navigation with SPLS was crucial to achieving a safe and effective outcome.

In this patient, the presence of CTVH introduced unique challenges to surgical planning and execution.^[17]^ The complete reversal of abdominal organ positions, with the liver located on the left side and the spleen on the right, required meticulous preoperative planning and intraoperative navigation. The altered anatomy could have complicated the identification of critical structures, such as the cystic duct and cystic artery, increasing the risk of intraoperative complications. Without advanced imaging and surgical techniques, managing such cases would be significantly more challenging.

The surgical management of patients with CTVH presents well-documented challenges, necessitating tailored approaches for procedures like cholecystectomy. Existing literature on cholecystectomy in CTVH underscores that conventional laparoscopic techniques require significant cognitive adjustment and mirror-image modification of port placement to navigate the reversed anatomy successfully.^[18]^ This established approach, while feasible, places a substantial burden on the surgeon’s spatial reasoning and does not objectively mitigate the risk within the altered Calot’s triangle of misidentifying critical structures. The diagnostic and therapeutic difficulties posed by anomalous anatomy are further echoed in reports of other acute abdominal conditions, such as appendicitis, where laparoscopy is valued for its ability to clarify ambiguous clinical pictures.^[19,20]^ Building upon this foundational understanding, our case introduces a significant technical evolution. We moved beyond reliance on cognitive compensation alone by integrating 2 advanced technologies: the single central umbilical port of SPLS, which simplified access and reduced spatial disorientation, and ICG fluorescence navigation, which provided an unambiguous, real-time map of the biliary anatomy. This synergistic combination represents a novel paradigm for achieving the highest standards of safety and precision in these extraordinarily complex scenarios.

In this complex case of CTVH, SPLS presented several potential benefits. Among these, this minimally invasive approach reduces surgical trauma by utilizing a single small incision, minimizing postoperative pain and complications such as incisional hernias.^[21]^ The umbilical incision not only facilitated faster recovery and reduced postoperative pain but also aligned with modern aesthetic standards, minimizing visible scarring.^[22]^ For this patient with CTVH, SPLS presented a unique navigational solution. The single-port system conceivably reduced the complexity of port placement in the setting of complete CTVH. Moreover, the anticipated “chopstick effect”^[23]^ was notably mitigated. One plausible explanation is that the reversed anatomy itself altered the instrument-to-target geometry, serendipitously promoting a more direct and effective dissection plane. This case highlights how specific anatomical variations can influence the applicability and performance of a surgical approach.

This experience underscores that the applicability and potential benefits of SPLS are highly context-dependent. In this unique setting of CTVH, its inherent limitations were less pronounced, while its minimally invasive and cosmetic advantages were fully preserved.

ICG fluorescence navigation played a critical role in ensuring procedural safety and precision. By administering ICG intravenously, we achieved real-time visualization of the biliary structures, functioning as an “intraoperative GPS.^[24]^” This technology was particularly valuable in the context of CTVH, where the reversed anatomy could have complicated manual identification of critical structures like the cystic duct and cystic artery. The fluorescence imaging provided a clear anatomical roadmap, facilitating accurate dissection of Calot triangle and reducing the risk of bile duct injury. This technology was particularly advantageous in navigating the mirrored anatomy. Nonetheless, the fundamental principles of laparoscopic cholecystectomy – such as meticulous dissection to achieve the Critical View of Safety and a readiness to convert to an open procedure when anatomy is unclear – remain the universal cornerstone of safety. Therefore, in resource-limited settings without ICG, strict adherence to these principles is paramount for managing such complex cases.

The integration of SPLS with ICG fluorescence navigation resulted in a synergistic effect – minimizing invasiveness while maximizing safety and precision. While this combined approach facilitated an excellent early recovery, as reflected in the rapid improvement of QoR-15 scores, future studies would benefit from standardized assessments at later time points (e.g., 2 weeks) to further elucidate the sustainability of recovery benefits. The minimally invasive approach of SPLS, combined with the precision of fluorescence navigation, ensured a streamlined and safe operating experience. The reduction in postoperative pain and complications, together with enhanced procedural accuracy, underscored the value of adopting these technologies. Additionally, the aesthetic advantage of the umbilical incision aligned with patient expectations for minimal scarring and cosmetically pleasing outcomes, reflecting modern surgical trends that prioritize both functional outcomes and patient satisfaction.

## 4. Conclusions

In conclusion, this case report highlights the successful synergistic application of SPLS and ICG fluorescence navigation in managing gallbladder calculus in a patient with CTVH. The integration of these advanced surgical techniques not only addressed the anatomical challenges posed by CTVH but also contributed to an optimal surgical outcome. This case underscores the importance of adopting innovative technologies in surgical practice and serves as a valuable reference for future clinical management of similar cases.

## 5. Patient perspective

Through a patient-centered approach, the treatment team maintained open communication with the patient, proactively eliciting and addressing the patient’s perspectives and feelings regarding the treatment. The general content is as follows: As a patient with CTVH, I was initially deeply concerned about the complexity and risks of the required surgery. However, the treatment team consistently engaged with me through a patient-centered approach. They actively listened to my feelings and priorities before explaining how ICG fluorescence navigation would improve safety by visually clarifying the biliary anatomy. They also recommended a single-port laparoscopic procedure, emphasizing that the single incision hidden in the umbilicus would align with my desire for both safety and minimal scarring. The surgery proceeded smoothly, and my recovery was uneventful. I was discharged on the third postoperative day with barely visible scarring. I am profoundly grateful for a treatment plan that respected both my clinical condition and personal values, resulting in an outcome that surpassed my expectations.

## Author contributions

**Conceptualization:** Dake Liu, Jianbin Gu, Fei Xue, Yubin Zhang, Jionghui Fu.

**Data curation:** Dake Liu, Weibo Liu, Jianbin Gu, Fei Xue, Yubin Zhang, Jionghui Fu.

**Formal analysis:** Dake Liu, Weibo Liu, Jianbin Gu, Fei Xue, Yubin Zhang.

**Investigation:** Jionghui Fu.

**Methodology:** Dake Liu, Weibo Liu, Jianbin Gu, Fei Xue, Yubin Zhang, Jionghui Fu.

**Writing – original draft:** Dake Liu.

**Writing – review & editing:** Dake Liu, Weibo Liu, Jianbin Gu, Fei Xue, Yubin Zhang, Jionghui Fu.
